# Attenuating the rate of total body fat accumulation and alleviating liver damage by oral administration of vitamin D-enriched edible mushrooms in a diet-induced obesity murine model is mediated by an anti-inflammatory paradigm shift

**DOI:** 10.1186/s12876-017-0688-4

**Published:** 2017-11-28

**Authors:** A. Drori, D. Rotnemer-Golinkin, S. Avni, A. Drori, O. Danay, D. Levanon, J. Tam, L. Zolotarev, Y. Ilan

**Affiliations:** 1 Gastroenterology and Liver Units, Department of Medicine, Hadassah-Hebrew University Medical Center, P.O.B 12000, -91120 Jerusalem, IL Israel; 2 Migal, Galilee Research Institute, Kiryat Shmona, Israel; 3 Obesity and Metabolism Laboratory, The Institute for Drug Research, School of Pharmacy, Faculty of Medicine, The Hebrew University of Jerusalem, Jerusalem, Israel

**Keywords:** Vitamin D, *Lentinula edodes*, Shiitake, NASH

## Abstract

**Background:**

Hypovitaminosis D is associated with many features of the metabolic syndrome, including non-alcoholic fatty liver disease. Vitamin D-enriched mushrooms extracts exert a synergistic anti-inflammatory effect. The aim of the present study is to determine the immunomodulatory effect of oral administration of vitamin D-enriched mushrooms extracts on high-fat diet (HFD) animal model of non-alcoholic steatohepatitis (NASH).

**Methods:**

C57BL/6 mice on HFD were orally administered with vitamin D supplement, *Lentinula edodes* (LE) mushrooms extract, or vitamin D-enriched mushrooms extract for 25 weeks. Mice were studied for the effect of the treatment on the immune system, liver functions and histology, insulin resistance and lipid profile.

**Results:**

Treatment with vitamin D-enriched LE extracts was associated with significant attenuation of the rate of total body fat accumulation, along with a decrease in hepatic fat content as measured by an EchoMRI. Significant alleviation of liver damage manifested by a marked decrease in ALT, and AST serum levels (from 900 and 1021 U/L in the control group to 313 and 340; 294 and 292; and 366 and 321 U/L for ALT and AST, in Vit D, LE and LE + Vit D treated groups, respectively). A corresponding effect on hepatocyte ballooning were also noted. A significant decrease in serum triglycerides (from 103 to 75, 69 and 72 mg/dL), total cholesterol (from 267 to 160, 157 and 184 mg/dL), and LDL cholesterol (from 193 mg/dL to 133, 115 and 124 mg/dL) along with an increase in the HDL/LDL ratio, and improved glucose levels were documented. These beneficial effects were associated with a systemic immunomodulatory effect associated with an increased CD4/CD8 lymphocyte ratio (from 1.38 in the control group to 1.69, 1.71 and 1.63), and a pro- to an anti-inflammatory cytokine shift.

**Conclusions:**

Oral administration of vitamin-D enriched mushrooms extracts exerts an immune modulatory hepato-protective effect in NASH model.

## Background

Non-alcoholic fatty liver disease (NAFLD) is the most common form of chronic liver disease in western countries [1]. The immune system plays an important role in the pathogenesis of the liver damage as well as in the development of liver fibrosis [2–5]. Epidemiologic data show that NAFLD and vitamin D deficiency often coexist [6], and both conditions are considered as cardio-metabolic risk factors [7]. Several studies have linked vitamin D, NAFLD, and diabetes [8]. The hypovitaminosis D is associated with central obesity, impaired glucose homeostasis, insulin resistance, hypertension, and dyslipidemia [8]. High serum levels of 25(OH)D3 were shown to protect against the development of NAFLD, and a negative correlation was shown between vitamin D levels and visceral fat area [9]. Vitamin D was found earlier to possess an anti-fibrotic property in the onset of fibrosis in specific genotypes for vitamin D receptor (VDR) [10].

Vitamin D through its active form 1α-25-dihydroxyvitamin D [1,25(OH)2D] is a secosteroid hormone that plays a key role in mineral metabolism [6]. Recent data suggest its role in immune regulation [11, 12]. Biologically active vitamin D, 1,25-dihydroxylvitamin D3, is synthesized by the classic two-step hydroxylation in the liver and kidneys. The 1,25-dihydroxylvitamin D3 can also be produced locally by immune cells in response to infection [13]. Several immune regulatory function of vitamin D were shown to include the induction of antimicrobial peptides, suppression of innate immune response, induction of Th2 cytokines, and promotion of T-regulatory T cells (Tregs) [13].

Hypovitaminosis D is common worldwide with a prevalence of 30% to 50%. This is mainly attributed to inadequate exposure to ultraviolet radiation and insufficient consumption of the vitamin [8]. An association between hypovitaminosis D and the metabolic syndrome has been described earlier. Patients with a serum 25-hydroxy vitamin D concentration < 10 ng/mL had an increased risk of abdominal obesity and a higher prevalence of the metabolic syndrome. In an intervention program, weight loss was strongly related to increased serum vitamin D concentration [14].

Extracts derived from *Lentinula edodes* (LE, Shiitake) edible mushroom exert an anti-inflammatory effect in animal models of immune-mediated colitis [15]. Mushrooms are an abundant source of ergosterol, which is the precursor of vitamin D_2_. Ergosterol converts into ergocalciferol (vitamin D_2_) following the exposure to ultraviolet (UV) light. Then, after ingestion and absorption, it goes through hydroxylation into the active form 25-hydroxyvitamin D [25(OH)D]. Recently, the vitamin D_2_-enriched mushrooms were studied, in order to verify the impact of both ingredients [16].

The aim of the present study is to determine the immunomodulatory effect of oral administration of vitamin D-enriched mushrooms extracts, and to assess its corresponding clinical effect on fatty liver disease and the related insulin resistance in the high-fat diet (HFD) animal model of non-alcoholic steatohepatitis (NASH).

## Methods

### Animals and experimental design

Experiments were carried out on animals according to the guidelines of the Hebrew University-Hadassah Institutional Committee for the Care and Use of Laboratory Animals with the committee’s approval. 10 weeks old male C57BL/6 mice were obtained from Harlan Laboratories (Jerusalem, Israel) and maintained in the Animal Core Facility of the Hadassah-Hebrew University Medical School. The mice were weighted weekly and fed in a liberal, restriction-free, commercially available HFD (Harlan, TD88137; 42% of the calories are from fat). Four groups of mice (*n* = 6, each) were orally treated three times a week for 25 weeks with one of the following: Group A (Control), saline (0.9% NaCl); Group B (Vitamin D), 25 μL of commercially available, over-the-counter, vitamin D supplement, containing 400 IU per drop, diluted at commercially available, over-the-shelf, ready-to-use, locally-made olive oil, equal to 10 UI per mouse per feed; Group C (LE), 25 μL of LE mushrooms extract containing 8.3 mg of dried mushroom, suspended in double-distilled water (DDW), per mouse per feed; Group D (LE + Vitamin D), 25 μL of vitamin D-enriched LE mushrooms extract containing 8.3 mg of dried mushroom and 10 IU for vitamin D, suspended in DDW, per mouse per feed. Mice were sacrificed using anesthetics.

### Preparation of vitamin D-enriched extract

Fruit bodies of LE at different development stages were picked. Immediately after picking the fruit bodies, they were exposed, post-harvest, to short pulses of UV-B irradiation in order to raise their vitamin D_2_ content [16]. The highest vitamin content was found in the fruit bodies that were picked at the “flat” development stage, two days following the optimal marketing picking time. The mushrooms were frozen-dried, milled to powder, and their vitamin D_2_ contents (on dry weight basis) were measured by high-performance liquid chromatography (HPLC) (MIGAL labs, Israel).

### Effect of UV-B exposure on shiitake Ergosterol, vitamin D_2_ and glucan content

As shown in Table 1, Shiitake mushroom’s Ergosterol and α-Glucans concentrations were not significantly altered following radiation exposure. However, Vitamin D_2_ content was increased significantly following UV-B exposure from a negligible concentration to 42.96 ± 7.21 μg/g DM (*p* value <0.05). Furthermore, in response to UV-B exposure Shiitake β-Glucan and total-Glucan concentrations were decreased significantly by 30% and 27.5%, respectively (*p* value <0.05).

**Table 1:** Active components in radiated (LE + D) and unirradiated (LE) *L. edodes* mushrooms

	LE (*n* = 3)		LE + D (*n* = 3)		
	M	SD	M	SD	*p* value
Ergosterol (mg/1 g DM)	1.98	0.84	1.24	0.70	0.327
Vit. D_2_ (μg/1 g DM)	ND	ND	42.96	7.21	0.0005
β-Glucan (% w/w)	22.35	1.01	15.80	0.88	0.0023
α-Glucan (% w/w)	1.3	0.04	1.38	0.07	0.2019
total-Glucan (% w/w)	23.64	1	17.18	0.81	0.0021

### Mushroom culture

Mushrooms were not exposed to UV-B along the growing process. LE mushrooms (S61 var., Fungisem) were grown on sterilized 3:2 mixture of eucalyptus sawdust and olive mill cake. Mixture was wetted to 60% water content and packed into Unicorn Type M filter polypropylene bags. Bags were autoclaved at 121 °C for 1 h, and cooled to 25 °C for inoculation with the spawn. Culture was incubated at 25 °C for 21 days. For fruiting, the temperature was reduced to 16 °C with a relative humidity of 90%, daily illumination for 12 h by fluorescent 500 lx “Daylight” and air CO_2_ concentration of 600–800 ppm [17].

### Irradiation procedure

A LH-840 (Xenon Corporation, Wilmington, MA) was used for pulsed UV light exposure. A 16″ linear B-type lamp was used (240 nm, No ozone generated). The LE mushrooms were exposed to 10 UV-B radiation pulses, at doses of 507 J/pulse at room temperature. The non-radiated and irradiated mushrooms were separately freeze-dried (Dr. Golik Co.), grind by mortar and pestle with liquid nitrogen, and then stored at −20 °C until analysis.

### Analysis of the Ergosterol and vitamin D_2_

The compounds of the vitamin D and Ergosterol fraction were extracted as described previously with minor modifications [17]. Their analysis was performed by UHPLC (Ultimate 3000, Thermo Scientific, MA, USA) coupled with diode array detector (DAD). The chromatographic separation was conducted on a C18 column (Aqua 3u C18 125A New Column 150 × 4.6 mm, Silicol). The sampler oven temperature was set to 4 °C, while the column oven temperature was set to 15 °C, with injection volume of 20 μL and flow rate at 1 mL/min. The separation was isocratic plan with 75% methanol: 25% acetonitrile. UV detection was performed in 210, 250, 265 and 280 nm. Ergosterol, vitamin D_3_ (Internal Standard) and vitamin D_2_ were determined by comparing the retention times of standards (ergosterol, cholecalciferol and ergocalciferol, Sigma Chemicals, Steinheim, Germany), and quantification was done by using a calibration curve.

### Total glucans, β-glucans and α-glucans analysis

The glucan concentrations were evaluated by using a Glucan Assay Kit (Megazyme® International Ireland Ltd., Bray Co, Wicklow, Ireland), based on a colorimetric reaction, according to the manufacturer’s instructions. Absorbance was measured at 510 nm using Ultrospec 2100 pro UV/Visible Spectrophotometer (Amersham Bioscience, Freiburg, Germany) against the GOPOD reagent blank and unknowns were compared to a glucose standard to calculate percent of glucan. Total Glucan (% *w*/w) and α-Glucan (% w/w) were measured and the difference between those two was calculated as the β-glucan (% w/w).

### Isolation of splenocytes

Spleens were kept in RPMI-1640 supplemented with FCS 10%. Spleens were crushed through a 70 μm nylon cell strainer [18] and centrifuged (1250 rpm for 7 min) to remove debris. Red blood cells were lysed. Splenocytes were suspended in 1 mL of fluorescence-activated cell sorting (FACS) buffer. Viability was assessed using trypan blue staining and was above 90%.

### FACS analysis

Flow cytometry was performed on splenocytes lymphocytes with antibodies for CD4, CD8, CD25 (eBioscience, San Diego, CA, USA) and NK1.1 (Biogem, Westlake village, CA, USA) epitopes using the LSR-II. Analysis was performed using FSC express software.

### Cytokine measurement

Serum interleukin 1-α(IL-1_α_), IL-1_β_, IL-4, IL-6, IL-10, IL-12, IL-13 IL-17, tumor necrosis factor alpha (TNF_α_) and interferon gamma (IFN_γ_) levels were measured in each animal using Custom Q-plex-10plex ELISA-based Chemiluminescent assay (Quansys Biosciences, Logan, UT, USA). Transforming growth factor beta (TGF_β_) levels were measured in each animal using Quantikine ELISA Mouse/Rat/Porcine/Canine TGF-b1 (R&D Systems, Minneapolis, MN, USA).

### Biochemistry analysis

Blood was collected from individual mice at euthanasia and serum aspartate aminotransferase (AST), alanine aminotransferase (ALT) and gamma-glutamyl transferase (gGT) levels were determined using Reflotvet Plus (Roche). Serum triglyceride (TG), total cholesterol (T-chol) and high-density lipoprotein (HDL) levels were measured using the Cobas®C 111 analyzer (Roche, Switzerland). Tail-end venous blood glucose levels were measured bi-weekly using Accu-Check Performa Tests (Roche). Low-density lipoproteins (LDL-c, was calculated by (0.9xT-Chol)-(0.9xTG/5)-28) at the end of the study [19].

### Body and liver fat content

The total in vivo body and ex vivo liver fat contents were evaluated by using the EchoMRI™-100H (EchoMRI, TX) at weeks 7 and 25 and after sacrifice, respectively.

### Liver histology

4–5 μm paraffin-embedded liver sections were prepared from each mouse and stained with hematoxylin-eosin (H&E). An unsighted pathologist examined the tissues using a light microscopy to score for morphological and histopathological changes that are characteristic of NAFLD Activity Score (NAS). The maximal score for steatosis (=3) was assigned for greater than 66%. The maximal score for lobular inflammation (=3) was assigned for >4 foci/200×, and hepatocyte ballooning (=2) was assigned for many cells/prominent ballooning. The maximal NAS score is a simple arithmetic combination of all three features (min. 0, max. 8) [20]. In addition, fibrosis was evaluated and semi-quantified (score 0–4).

### Hepatic triglyceride (hTG) content

Accumulation of intracellular TGs within the liver was quantified using a modification of the Folch method [21]. hTGs were extracted from aliquots of snap-frozen livers and then assayed using a GPO-Trinder kit (Sigma, Israel), and the levels were normalized to per gram of liver tissue in the homogenate.

### Statistical analysis

Statistical analysis was performed using Kruskal-Wallis test and Mann-Whitney (only if the former showed statistical significance, *p* value <0.05) (Using GraphPad Prism 6.01). Standard Error (SE) is indicated by error bars at all figures.

## Results

### Effect of treatment on metabolic parameters

**Fig. 1:**
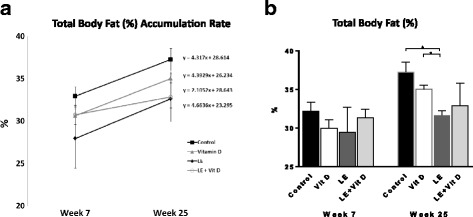
Effect of oral administration of vitamin D-enriched mushrooms on liver damage. Trend-lines representing the rate of total body fat (%) accumulation, calculated as the slope of the linear line throughout the study (**a**). Effect of treatment on total body fat (%) as measured by EchoMRI at the end of the experiment (**b**). Data represent mean +/− SE from *N* = 4–6 mice per group. *p* value (by Kruskal-Wallis test) < 0.05 for Total body fat (%). * - *p* value <0.05 (by Mann-Whitney test)

The aim of the study was to determine the immunomodulatory effect of oral administration of vitamin D-enriched mushrooms extracts, and to assess its corresponding clinical effect on fatty liver disease and the related insulin resistance in the HFD model of NASH. Treatment with LE + vitamin D significantly attenuated the rate of body fat accumulation as calculated by the trend-lines and their slope [Fig. 1a].

As noted, the slope decreases from 4.31, 4.39 and 4.66 at Control, Vitamin D and LE groups to 2.10 at the LE + vitamin D group. At week 25, there was a decrease in the percentage of total body fat between the control group and all three treatment groups [Fig. 1b].

**Fig. 2:**
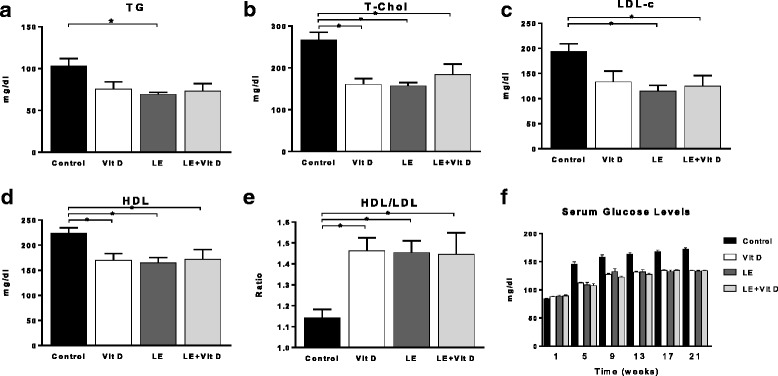
Effect of oral administration of vitamin D-enriched mushrooms on serum lipids & glucose levels. Effect of treatment on triglycerides (TG), total cholesterol (T-Chol), high-density lipoproteins (HDL), low-density lipoproteins (LDL-c, calculated) and HDL/LDL ratio (**a-e**). Effect of treatment on serum glucose levels (**f**). Data represent mean +/− SE from *N* = 4–6 mice per group. *p* value (by Kruskal-Wallis test) < 0.05 for all measured parameters. * - *p* value <0.05 (by Mann-Whitney test)

The TG levels, T-Chol and LDL-c were decreased in all treatment groups, compared to the control group [Fig. 2a-d], while the ratio between serum HDL and LDL-c was increased at all treatment group [Fig. 2e].

Starting from week 3 (data not shown), and throughout all of the experiment, the three treatment groups showed a statistically significant decrease in the average serum glucose levels, compared to the control group. No significant differences were noted between the average serum glucose levels for three treatment groups, except at anecdotal point - week 11 (data not shown), [Fig. 2f].

Treatment was also associated with a significant decrease in body weight. For example, at week 25, the average weight of the control group was 47.4 g, compared with 40.6 g, 40.2 g and 40.5 g at the vitamin D, LE and LE + vitamin D treated groups, respectively (all *p* values <0.05. Data not shown).

### The effect of treatment on liver damage

**Fig. 3:**
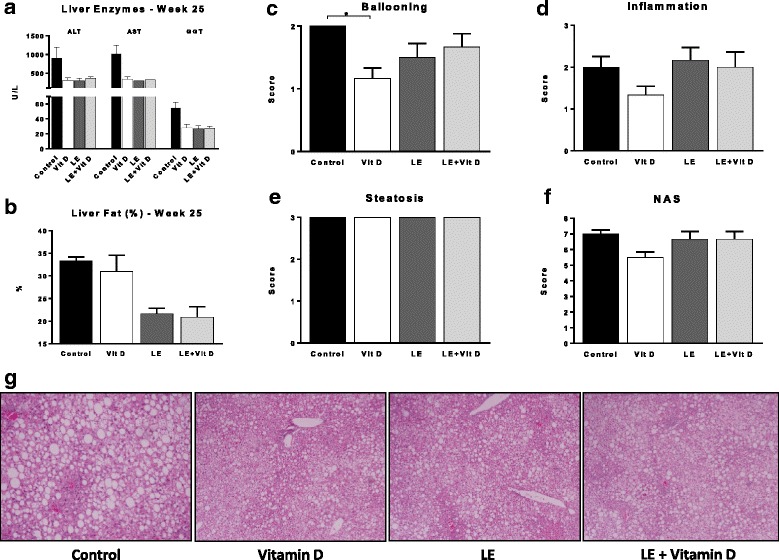
Effect of oral administration of vitamin D-enriched mushrooms on liver damage. Effect of treatment on serum liver enzymes levels: ALT, AST and GGT (**a**). Effect of treatment on hepatic fat content (%) as measured by EchoMRI (**b**). Effect of treatment on NAS score: ballooning, inflammation, Steatosis and overall NAS score were calculated for mice in all groups (**c-f**). Effect of treatment on liver histology: Representative slides from all groups are shown (H&E, ×10) (**g**). Data represent mean +/− SE from *N* = 4–6 mice per group. *p* value (by Kruskal-Wallis test) < 0.05 for Liver fat (%) week 25 and hepatocytes ballooning. * - *p* value <0.05 (by Mann-Whitney test)

A significant alleviation of the liver damage as manifested by a marked decrease in serum ALT, AST and GGT levels in all three treated groups at week 25, compared with the control group [Fig. 3a].

The hTG content did not change between the three treated groups and the control group.

A statistically significant decrease in the hepatic fat content (by Post-mortem Liver EchoMRI) was noted following treatment with mushrooms extracts compared to the control group. This effect was not achieved when treating with vitamin D alone [Fig. 3b].

Upon histological examination, a reduction was noted for hepatocytes ballooning score, and in the NAS score, in the Vitamin D group [Fig. 3c-f].

Representative photographs of liver sections (H&E, ×10) from all groups are shown in Fig. 3g. A reduction in the ballooning is noted in Vitamin D and LE groups, and a reduction in the inflammation was noted in Vitamin D and LE + Vitamin D treated groups. Photos courtesy of Dr. Areej A. S. Khatib, M.D., Bethlehem University.

### Effect of treatment on the immune system

**Fig. 4:**
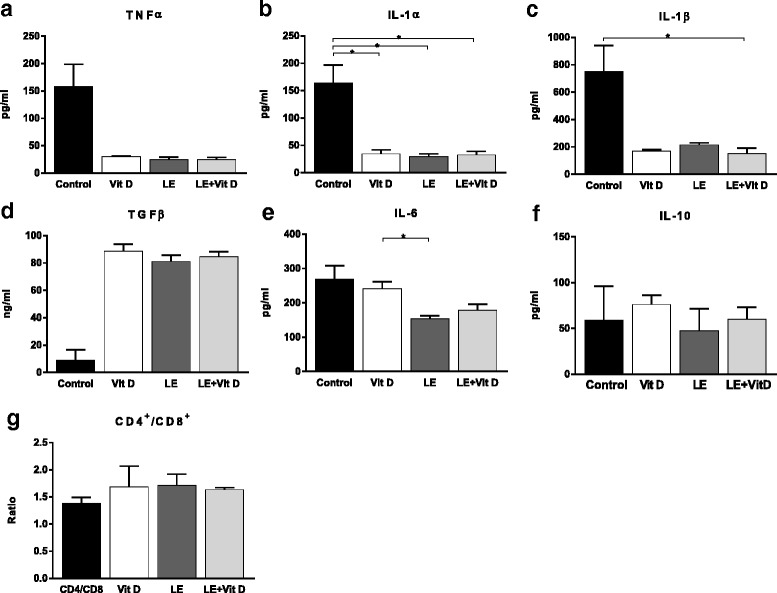
Effect of oral administration of vitamin D-enriched mushrooms on the immune system. Serum levels of TNF_α_, IL-1_α_, IL-1_β_, TGF_β_, IL-6 and IL-10 were measured by ELISA at the end of the study (**a**-**f**). FACS analysis was performed on lymphocytes isolated from spleens. Effect of treatment on the CD4/CD8 lymphocyte ratio (**g**). Data represent mean +/− SE from *N* = 4–6 mice per group. *p* value (by Kruskal-Wallis test) < 0.05 for TNF_α_, IL-1_α_, IL-1_β_ and IL-6. * - *p* value <0.05 (by Mann-Whitney test)

A significant reduction was noted in pro-inflammatory cytokines serum levels in all three treated groups - TNF_α_, IL-1_α_ and IL-1_β_. TGF_β_1 serum levels increased in all three treated groups, compared to the control group. A trend for an increase IL-10 was noted, following treatment with Vitamin D and LE + Vitamin D. The IL-10/TNF_α_ ratio and IL-4/TNF_α_ ratio were increased in all the treated groups, compared to control group (data not shown) [Fig. 4a-f].

The oral administration of vitamin D-enriched mushrooms exerted a systemic immune modulatory effect as noted by a trend for alteration of splenic lymphocytes sub-populations. A change in the CD4/CD8 lymphocyte ratio was noted with an increased ratio in all three treated groups, compared with the control group. No other significant changes were noted in other studied subsets of lymphocytes [Fig. 4g].

## Discussion

Much progress in the understanding of the pathophysiology of NASH has been made. However, there is still no approved therapy for this epidemic [1]. Several of the drugs being developed for NASH target fibrosis, and/or carry side effects. These may prohibit their long term use in patients with mild to moderate disease, when inflammation (and not fibrosis) underlines the liver damage [1]. The results of the present study show that vitamin-D enriched mushrooms extract exerts a beneficial immunomodulatory effect alleviating the liver damage and the metabolic parameters in the HFD model of NASH. Each of the compounds exerted a beneficial effect on their own. However, a synergistic effect was noted on body fat accumulation.

A pro-inflammatory status underlines the development of NASH. Immune modulatory treatments are being evaluated for NASH [3, 22–28]. A correlation between TNF_α_ and degree of severity of the liver disease were described [29, 30]. Adaptive immune responses triggered by TNF_α_-mediated oxidative stress contribute to hepatic inflammation in NASH [31]. Similarly, activation of kupffer cells enhances IL-1 production, contributing to hepatocyte dysfunction, necrosis, apoptosis, and generation of extracellular matrix proteins leading to fibrosis. IL-1 is also known to regulate hepatic steatosis [32]. Increased pro-IL-1_β_ levels correlate with disease severity [33]. Neutralization of IL-1 by IL-1 receptor antagonist (IL-1Ra) prevents liver injury [32]. Serum IL-1 receptor antagonist (IL-1RA) and liver mRNA expression of IL-1RN are associated with NASH and with the degree of lobular inflammation in liver [34]. In the present study, oral administration of vitamin D-enriched LE extracts exerted a systemic immune modulatory effect as noted by the alteration of splenic lymphocytes sub-populations. Treatment was also associated with a significant decrease in the serum levels TNF_α_, IL-1_α_, and IL-1_β_.

The role of TGF_β_ serum levels in the pathogenesis of NASH is somewhat controversial. Some studies showed lack of correlation with degree of severity of disease [35], while other suggested that TGF_β_ signaling pathway in hepatocytes contributes to hepatocyte death and lipid accumulation through *Smad* signaling and reactive oxygen species production that promote the development of NASH [36, 37]. In some clinical trials using immune modulatory agents in patients with NASH, an increase in TGF_β_ serum levels correlated with the beneficial effect of the drug [26]. In the present study, an increase in TGF_β_ serum levels was noted in all treated groups. Being a multifactorial disease, NASH is also multiple cytokine-mediated disorder [38, 39].

The immune modulatory effects were associated with a significant alleviation of the liver damage manifested by a marked decrease in ALT, AST, and GGT serum levels in all three treatment groups. A corresponding decrease in hepatic fat content and attenuation of body fat accumulation is established. A significant decrease in serum TGs, T-Chol, along with an increase in the HDL/LDL ratio, and improved glucose levels, were noted in all treated groups.

The vitamin D-enriched LE extract described in the present study was previously shown to have a potent anti-inflammatory effect. Oral administration of LE extracts is known to alleviate immune-mediated colitis [15]. The effect is associated with altered NKT regulatory lymphocyte distribution and increased intrahepatic CD8+ T lymphocyte trapping.

Treatment with vitamin D_2_-enriched mushrooms extracts alleviates Concanavalin A- immune-mediated liver injury [16]. Following feeding of the vitamin D-enriched mushrooms extracts to immune-mediated hepatitis harboring mice, ALT serum levels are decreased and proportion of severe liver injury is declined. A corresponding histological improvement of the immune mediated liver injury is also noted in treated mice. The data showed a synergistic effect between the anti-inflammatory effect of the mushroom extracts and that of the vitamin D [16]. In the present study, oral administration of vitamin D-enriched LE extracts had a beneficial effect on all tested immune, liver, and metabolic parameters, while the administration of non-vitamin enriched extracts or vitamin D alone exerted an effect only on some of these endpoints. A synergistic effect for the vitamin D-enriched LE extract was noted for the rate of body fat accumulation.

Mushrooms-associated immunomodulatory polysaccharides, such as Glucans (α-Glucans and β-Glucans) contribute to their anti-inflammatory effect [40]. Innate immune cells express pattern recognition receptors (PRRs) including dectin-1, Toll-like receptors, and mannose receptors on their cell surfaces. These PRRs recognize pathogens by binding to highly conserved pathogen-associated molecular patterns (PAMPS) such as beta-glucan, mannan, and lipopolysaccharide. Binding of β-glucans to dectin-1 expressed by macrophages or dendritic cells leads to innate cells activation of adaptive immune cells via secretion of interleukins (IL-4, IL-6) and TNF_α_ [40]. In vitro, the immune effect of β-glucans was dependent of their structure, molecular weight and compositional characteristics [41]. β-D-glucan manifested an immunomodulatory activity on THP-1 macrophages, inhibited the inflammatory phase of nociception, and reduced the number of total leukocytes and myeloperoxidase levels induced by LPS, supporting their anti-inflammatory activity [42].

Hypovitaminosis D is associated with NAFLD, increased insulin resistance, impaired insulin secretion, and is related to type 2 diabetes mellitus (T2DM) [43]. Local vitamin D signaling regulates hepatic and pancreatic islet functions contributing to both hepatic insulin sensitivity and islet insulin secretion [43]. Studies have suggested the benefits for vitamin D maintenance, or dietary manipulation, for prevention and treatment of obesity-induced T2DM and NAFLD. A recent cross-sectional study evaluated the correlation between NAFLD and vitamin D in 5000 men and women [44]. Decreased vitamin D levels were associated with an increased risk of NAFLD. Vitamin D was found to be an independent factor for NAFLD prevalence, implying that vitamin D interventional treatment may control the disease [44]. In a NASH animal model of choline-deficient diet, 1,25-vitamin D3 supplement slowed the development and progression of NASH [45]. Administration of 1,25-vitamin D3 decreased free fatty acids, triglycerides, thiobarbituric acid-reactive substances, number of apoptotic cells, expression of tissue inhibitor of metalloproteinase-1,and CK18-M30 in the liver, and improved liver histology. No change was noted in total antioxidant capacity of the liver [45, 46].

A recent 239 patient’s trial showed that plasma vitamin D levels are not associated with insulin resistance, amount of liver fat accumulation, or the severity of NASH [47]. A 398 patient’s trial showed that low levels of 25-OH vitamin D were not independently associated with liver damage in morbidly obese patients with NAFLD [48]. However, 25-OH vitamin D levels were inversely correlated to NAS biopsy score and steatosis. Vitamin D levels were lower in patients with significant fibrosis [47]. This discrepancy may be explained by population differences, and other confounding factors which were not accounted for. In addition, the beneficial effect of vitamin D-enriched mushrooms in NASH, as noted in the present study, may be due to a direct synergistic effect of the mushrooms extract and vitamin D, independent of baseline vitamin D serum levels.

The exact mechanism in which either vitamin D or LE mushrooms extracts exert all the above beneficial effects is still unknown. The fact that some beneficial effects were achieved by the Vitamin D and other effects were achieved by the LE might suggest a different biochemical mechanism, with a synergistic effect of both pathways. Another study, in an attempt to decipher these mechanisms and isolate the active ingredient (or ingredients) of LE, is underway.

## Conclusions

In summary, the data of the present study supports a beneficial effect of oral administration of LE extracts enriched with vitamin D in alleviating the liver damage, and insulin resistance in a mouse model of NAFLD. A synergistic effect was noted on body fat accumulation. Considering the high safety profile of these extracts, the data supports their potential use in patients with early-stage NASH.

## Abbreviations

(U)HPLC: (Ultra-) High-Performance Liquid Chromatography
ALT: Alanine aminotransferase
AST: Aspartate aminotransferase
DDW: Double-Distilled Water
DM: Dried Mushroom
GGT: Gamma-Glutamyl Transferase (gGT)
HDL: High Density Lipoprotein
HFD: High-Fat Diet
IFN_γ_: Interferon gamma
IL: Interleukin
IL-1Ra: IL-1 receptor antagonist
IL-1RA: Interleukin 1 receptor antagonist
LE, Shiitake: *Lentinula edodes*
NAFLD: Nonalcoholic Fatty Liver Disease
NAS: NAFLD Activity Score
NASH: Nonalcoholic Steatohepatitis
PAMPS: Pathogen-associated Molecular Patterns
PRRs: Pattern Recognition Receptors
TG: Triglycerides
TGF_β_: Transforming Growth Factor beta
TNF_α_: Tumor Necrosis Factor alpha
Tregs: T-regulatory T cells
VDR: Vitamin D receptor
